# CRISPR/Cas9-generated mouse model of Duchenne muscular dystrophy recapitulating a newly identified large 430 kb deletion in the human *DMD* gene

**DOI:** 10.1242/dmm.037655

**Published:** 2019-04-25

**Authors:** Tatiana V. Egorova, Evgenia D. Zotova, Denis A. Reshetov, Anna V. Polikarpova, Svetlana G. Vassilieva, Dmitry V. Vlodavets, Alexey A. Gavrilov, Sergey V. Ulianov, Vladimir L. Buchman, Alexei V. Deykin

**Affiliations:** 1 Laboratory of Modeling and Gene Therapy of Hereditary Diseases, Institute of Gene Biology, Russian Academy of Sciences, Moscow, 119334, Russia; 2 Marlin Biotech LLC, Moscow, 143026, Russia; 3 Research Centre for Genetic Medicine, Moscow, 117292, Russia; 4 Veltischev Scientific Research Clinical Paediatric Institute, Moscow, 125412, Russia; 5 Group of Genome Spatial Organization, Institute of Gene Biology, Russian Academy of Sciences, Moscow, 119334, Russia; 6 Laboratory of Structural and Functional Organization of Chromosomes, Institute of Gene Biology, Russian Academy of Sciences, Moscow, 119334, Russia; 7 Faculty of Biology, M.V. Lomonosov Moscow State University, Moscow, 119991, Russia; 8 School of Biosciences, Cardiff University, Cardiff, CF10 3AX, UK; 9 Core Facilities, Institute of Gene Biology, Russian Academy of Sciences, Moscow, 119334, Russia

**Keywords:** Exon skipping, Muscle degeneration, New deletion, Genome editing, DMD, Mouse model

## Abstract

Exon skipping is a promising strategy for Duchenne muscular dystrophy (DMD) disease-modifying therapy. To make this approach safe, ensuring that excluding one or more exons will restore the reading frame and that the resulting protein will retain critical functions of the full-length dystrophin protein is necessary. However, *in vivo* testing of the consequences of skipping exons that encode the N-terminal actin-binding domain (ABD) has been confounded by the absence of a relevant animal model. We created a mouse model of the disease recapitulating a novel human mutation, a large *de novo* deletion of exons 8-34 of the *DMD* gene, found in a Russian DMD patient. This mutation was achieved by deleting exons 8-34 of the X-linked mouse *Dmd* gene using CRISPR/Cas9 genome editing, which led to a reading frame shift and the absence of functional dystrophin production. Male mice carrying this deletion display several important signs of muscular dystrophy, including a gradual age-dependent decrease in muscle strength, increased creatine kinase, muscle fibrosis and central nucleation. The degrees of these changes are comparable to those observed in *mdx* mice, a standard laboratory model of DMD. This new model of DMD will be useful for validating therapies based on skipping exons that encode the N-terminal ABD and for improving our understanding of the role of the N-terminal domain and central rod domain in the biological function of dystrophin. Simultaneous skipping of exons 6 and 7 should restore the gene reading frame and lead to the production of a protein that might retain functionality despite the partial deletion of the ABD.

## Abstract (precis)

**Summary:** The authors describe the creation of a mouse strain that reproduces a newly identified deletion mutation in a DMD patient in Russia, and present the characteristics of this new model.

## INTRODUCTION

Duchenne muscular dystrophy (DMD) is an X-linked recessive disease affecting approximately 1 in 5000 newborn males (Mendell and Lloyd-Puryear, 2013). The disease is characterized by progressive muscle wasting, leading to patients becoming fully dependent on mobility aids early in life. The average lifespan of DMD patients is 20 years, and the major causes of death are respiratory failure and cardiac arrest due to weakness of the corresponding groups of muscles (Passamano et al., 2012). The disease is caused by the absence of the functional dystrophin protein in muscle cells. Dystrophin is a large (3685 amino acids in humans), evolutionarily conserved protein. It is a crucial component of a molecular complex, the main function of which is to ensure muscle membrane integrity and stability by playing the role of a ‘molecular spring’, connecting a cytoskeletal network to the sarcolemma and extracellular matrix. Dystrophin consists of four functional domains: the N-terminal actin-binding domain (ABD), the central rod domain containing 24 spectrin-like repeats and four proline-rich hinges, the cysteine-rich domain, and the C-terminal domain (Fig. 2B) connecting dystrophin to the membrane components of the dystrophin-associated glycoprotein complex (DAGC) and the syntrophin-dystrobrevin subcomplex (Gao and McNally, 2015; Le Rumeur, 2015). When dystrophin is absent or aberrant, the sarcolemma anchoring of actomyosin complexes becomes weaker, which effectively disrupts membrane integrity during muscle contraction and ultimately leads to myofibre necrosis and inflammation. Regeneration compensates for the loss of myofibres until the myoblast pool becomes depleted, which is associated with muscle degeneration and disease progression (Davies and Nowak, 2006).

Most cases of DMD are caused by frameshift-generating deletions of one or more exons of the *DMD* gene, whereas mutations that preserve the reading frame are the cause of a milder form of the disease known as Becker muscular dystrophy (BMD) (Koenig et al., 1989).

Several therapeutic strategies have been proposed for DMD treatment: premature stop codon ignoring (Aurino and Nigro, 2006; Malik et al., 2010); upregulating utrophin, a protein that can functionally substitute for dystrophin (Guiraud et al., 2015); cell therapy (Bajek et al., 2015); viral delivery of a shortened form of dystrophin (Le Guiner et al., 2015); and exon skipping with reading frame restoration via exclusion of several more exons at the splicing stage (Niks and Aartsma-Rus, 2017). Following successful clinical trials, an exon skipping procedure in which exon 51 is skipped has received accelerated approval from the US Food and Drug Administration (FDA); this intervention can be beneficial for ∼13% of DMD patients (Young and Pyle, 2016). To test the feasibility of exon skipping as a therapeutic approach for other genetic variants of DMD, creating animal models recapitulating the pathophysiological consequences of the disease-associated human mutations is necessary. The highly conserved sequence and exon-intron structure between human and mouse dystrophin-encoding genes make it possible to accurately reproduce human mutations by targeted modifications of the mouse genome. More than 50 mouse models with mutations in the *Dmd* gene have been produced and characterized. These models include 11 mouse lines with various single mutations in the gene and many variants of double or multiple knockouts (McGreevy et al., 2015). Most of the latter group is based on the *mdx* mouse model, which bears a premature stop codon within exon 23 of the mouse *Dmd* gene (Sicinski et al., 1989; McGreevy et al., 2015). However, there is only one model with an affected N-terminal ABD, encoded by exons 1-7 (McGreevy et al., 2015; Vulin et al., 2015).

Accurate modelling of many genetic variants of human DMD requires the introduction of large deletions into the mouse *Dmd* gene. CRISPR/Cas9 technology is an efficient gene-editing strategy for genome modifications, including the generation of such large deletions (Korablev et al., 2017). These large deletions can be created by simultaneously introducing two single-guide RNAs (sgRNAs) or sgRNA-encoding plasmids targeting sequences at the border of the fragment to be deleted together with a Cas9 enzyme source, i.e. an expression plasmid, mRNA or the recombinant protein itself, into the mouse zygote. This technology has been successfully used for the production of cell lines and mouse strains carrying large deletions. For example, CRISPR/Cas9 has been used to create: a 65 kb deletion in embryonic stem (ES) cells and mice zygotes by transfection or injection of two circular plasmids encoding sgRNAs and the Cas9 protein (Zhang et al., 2015); a 95 kb deletion in one-cell rodent embryos by co-injection of two sgRNAs with the Cas9 protein (Wang et al., 2015); and a 1 Mb deletion in human cell lines by transfection of sgRNA and Cas9-encoding plasmids (Essletzbichler et al., 2014).

In this study, we used the injection of two sgRNAs and *in vitro*-synthesized *Cas9* mRNA into mouse single-cell embryos to delete a 430 kb fragment of the dystrophin gene spanning exons 8-34. Using this approach, we produced a mouse model of a novel genetic variant of DMD that was recently identified in a Russian patient. This mouse model represents the largest deletion in the *Dmd* gene.

## RESULTS

### Identification of a new mutation in the *DMD* gene leading to the development of Duchenne muscular dystrophy

We identified a new mutation in the *DMD* gene of a male patient of Russian origin. The patient, now 8 years old, was born after a normal-term pregnancy in a family with no history of neuromuscular disorders. He began to walk independently at the age of 13 months and developed normally until the age of 5, when the first manifestations of muscle wasting and fatigue were observed by the parents. At that time, his physical examination at the Russian Children's Neuromuscular Center revealed no gait disturbances. The patient was actively running and could squat and rise, jump and hop on one or two legs. He did not use handrails and moved step by step on stairs when ascending or descending. No pathological features of the cranial nerves were observed. However, the patient had a slight stiffness of the ankle joints, scapular winging, calf pseudohypertrophy, macroglossia and slight hyperlordosis in his lumbar spine. The use of some Gowers' manoeuvres when rising from the floor was also observed. The patient's muscle tone was decreased, and his muscle strength was ∼4-5 on the Medical Research Council Paralysis Scale. The deep tendon reflexes were absent. During the 6 min walk test, which was repeated annually, the boy walked 453 metres in 2016 and 527 metres in 2017. Nevertheless, an electromyography (EMG) study revealed myogenic changes. Creatine kinase (CK) was markedly elevated to 20,823 U/l (normal is up to 190 U/l), which is consistent with a diagnosis of DMD. To confirm the diagnosis, a DNA diagnostic analysis was performed. A multiplex ligation-dependent probe amplification (MLPA) assay using genomic DNA from the patient showed the absence of the product from exon 8 to exon 34, which suggests a deletion in the *DMD* gene. To confirm the deletion and to identify its exact borders, whole-genome sequencing was performed. An ∼0.3 Mb region spanning exons 8-34 that corresponded to ∼12% of the *DMD* coding sequence was deleted from the patient's genome. The exact coordinates of the deletion in the Hg19 assembly were chromosome X (ChrX): 32389015-32717668.

**Fig. 1.**
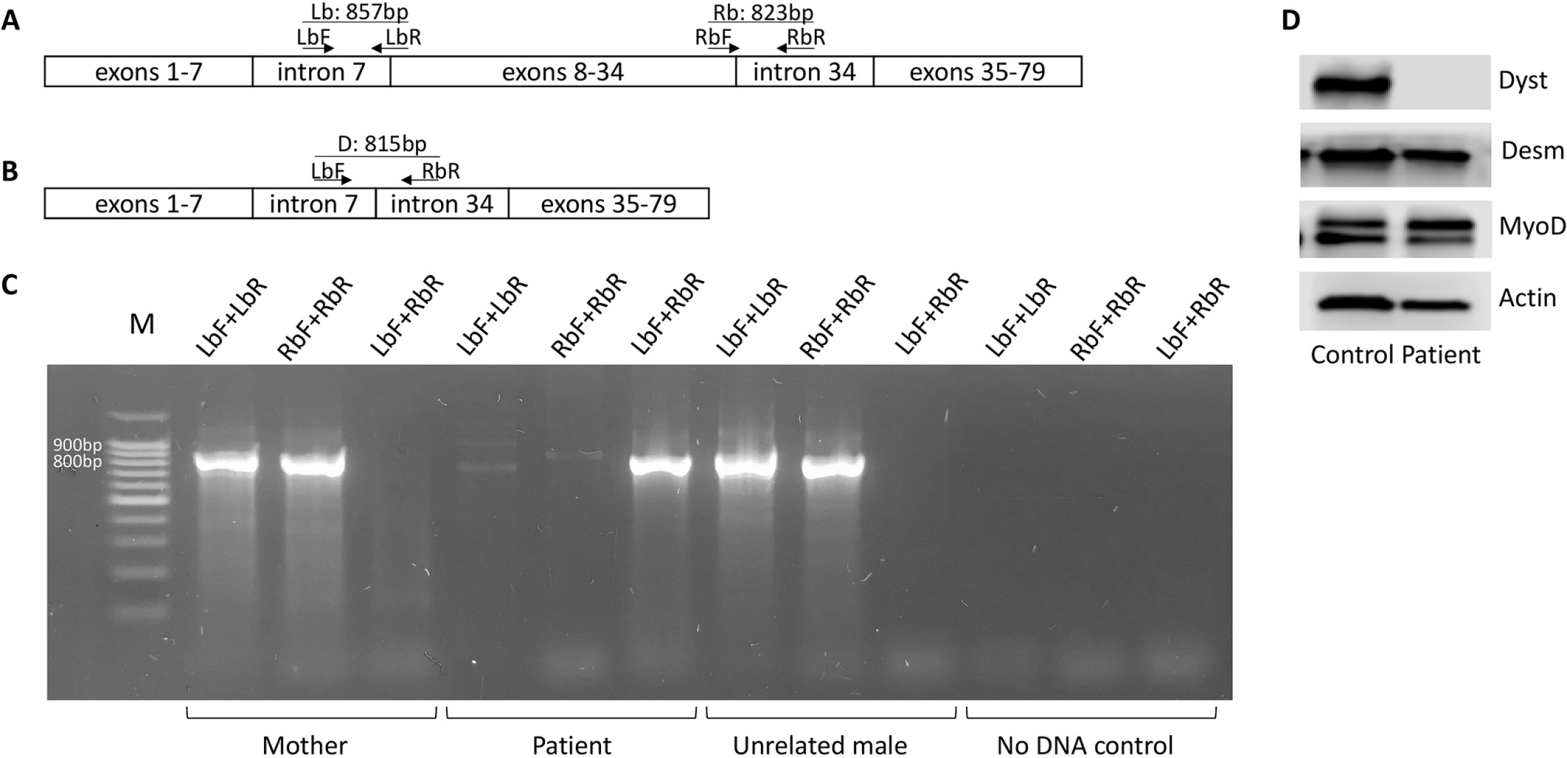
**Analysis of PCR products amplified with primers encompassing the deletion in the patient's *DMD* gene.** (A,B) The positions of primers within the normal (A) and mutated (B) genes (not drawn to scale). (C) Agarose gel of fragments amplified from the genomic DNA of the patient, his mother and an unrelated healthy male (as a negative control). The presence of an 857 bp product amplified with the LbF and LbR primers from genomic DNA of the mother and a healthy male indicates that the *DMD* gene is intact at the left border of the patient's deletion. Similarly, the presence of an 823 bp product amplified with the RbF and RbR primers indicates that the right border of the patient's deletion is intact in these DNAs. In contrast, no amplification products were obtained when the patient's genomic DNA was amplified with these two pairs of primers. A readthrough product of 815 bp was amplified using the LbF and RbR primers only from the genomic DNA of the patient. M–100 plus DNA ladder (NL002, Evrogen). (D) Western blot analysis of full-length dystrophin expression in myoblasts transdifferentiated from patient and control human fibroblasts using antibodies against the dystrophin C-terminus (ab15277). Transdifferentiation success was verified by staining the muscle cell markers desmin (ab15200) and MyoD (sc-304). Alpha-actin was used as a loading control. A representative image of three independent experiments is shown.

To examine whether the patient's mother was a carrier of the mutation in the *DMD* gene, polymerase chain reactions (PCRs) with four different primers (listed in Table S1) were performed using genomic DNA from the patient, the patient's mother and an unrelated healthy male (negative control). The primer pair LbF+LbR was used for amplification of a fragment spanning the beginning of the deletion, and RbF+RbR was used to amplify the end of the deletion, a combination of LbF+RbR allowed analysis of the deletion readthrough. Any PCR product from the primer sets LbF+LbR and RbF+RbR were expected to be amplified in only the absence of the deletion (Fig. 1A). In contrast, amplification with primers LbF+RbR would occur in only the presence of the deletion (Fig. 1B). The results (Fig. 1C) showed that the patient's mother had no deletion, which indicated the *de novo* acquisition of this mutation. Sanger sequencing of the corresponding PCR products confirmed the absence of the deletion in the mother's genome as well as its presence in the patient (data not shown).

Primary fibroblasts were sampled from the patient and transdifferentiated into myoblasts to assess full-length dystrophin expression, which was not detected using antibodies against the C-end of the protein (Fig. 1D).

### Guide selection for generation of the deletion in the mouse *DMD* gene

**Fig. 2.**
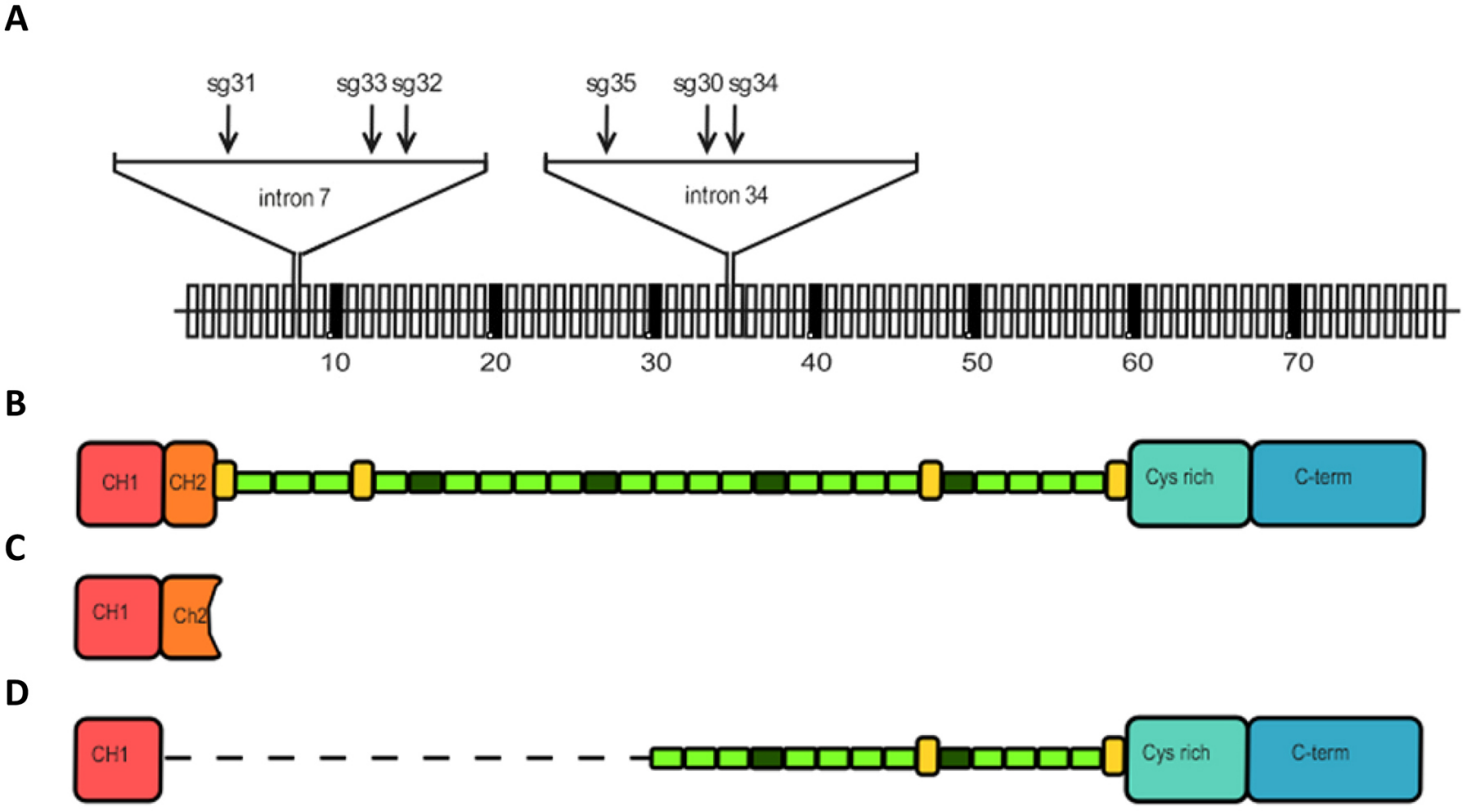
**Guide RNA positions on the gene.** (A) A scheme of the mouse *Dmd* gene exons (vertical boxes, not drawn to scale) and positions of sgRNAs selected for generation of a large deletion encompassing a region between introns 7 and 34. (B-D) Schemes of dystrophin protein domain organization: full-length dystrophin (B), theoretical shortened dystrophin in which expression is possible in muscle cells of patients with deletion of *DMD* exons 8-34 (C) and theoretical shortened dystrophin in which expression is possible in the muscle of patients after additional simultaneous skipping of exons 6 and 7 (D).

The CRISPR/Cas9 genome editing technique was employed to generate a mouse model recapitulating the modification of the *DMD* gene manifested in the patient's genome. Deletion boundaries in the *DMD* gene of the patient were located in the 7th and 34th introns. Therefore, to generate a deletion in the mouse genome, sgRNAs in the corresponding introns of the mouse *Dmd* gene were designed. Three sgRNAs within each of these introns were selected using the CHOPCHOP online search tool (Montague et al., 2014; Labun et al., 2016) (Table 1 and Fig. 2A). All selected sgRNAs were predicted to have no off-target sites with less than three mismatches and were tested in a series of control experiments (Fig. S1). Each sgRNA was first tested *in vitro* on a circular recombinant plasmid containing a PCR-amplified target fragment from the mouse *Dmd* gene. For all selected guides, specific (digestion of the target fragment and lack of digestion of control fragments) and fairly efficient linearization of the plasmid DNA was observed in this test (Fig. S1). Further *ex vivo* tests were performed on blastocysts, first for each sgRNA individually to select the most promising guides, followed by pairwise testing. The scheme of the testing protocol with sgRNA 31 as an example is shown in Fig. S1, and the efficiency of cleavage for each sgRNA is shown in Table 1. A combination of sgRNA 31 for cutting within intron 7 and sgRNA 30 for cutting within intron 34 was chosen for the creation of the model. The efficiency of the target fragment deletion was estimated in seven experiments, in which 120 total embryos were injected with sgRNA 30+31 and the *Cas9* mRNA mix and cultivated up to the blastocyst stage, followed by PCR and sequence analysis of their DNA (Fig. S1). Deletions were identified in 43 embryos, which corresponded to ∼36% of all the injected embryos.

**Table 1.**
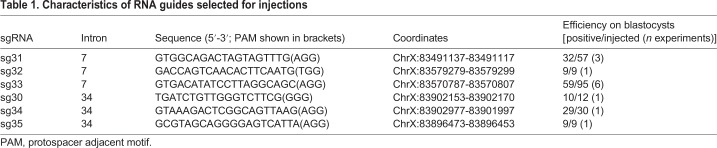
**Characteristics of RNA guides selected for injections**

### Production of mice with the targeted deletion

A total of 639 embryos were injected with the *Cas9* mRNA and sgRNA 30+31 mix and transplanted into 85 recipient mice in ten independent experiments. Among 94 newborns, 19 mice with the desired deletion were found, which corresponded to ∼20% of all newborns and 3% of all transplanted embryos. Deletion borders were identified by direct Sanger sequencing of the PCR products. Alignment to the theoretical sequence is shown in Fig. S2. Deletion borders for each guide are shown in Table 2. Taking the N nucleotide of the NGG PAM site as 0, the positions of these deletion borders spanned from nucleotide −10 to nucleotide −1 for sgRNA 30 and from nucleotide −3 to nucleotide +21 for sgRNA 31. In one case, the insertion of four nucleotides into the cleavage site was observed. In two female mice (#85 and #92), more than one modification in the gene was revealed, which could be explained by either different mutations in two X chromosomes or genetic mosaicism.

**Table 2.**
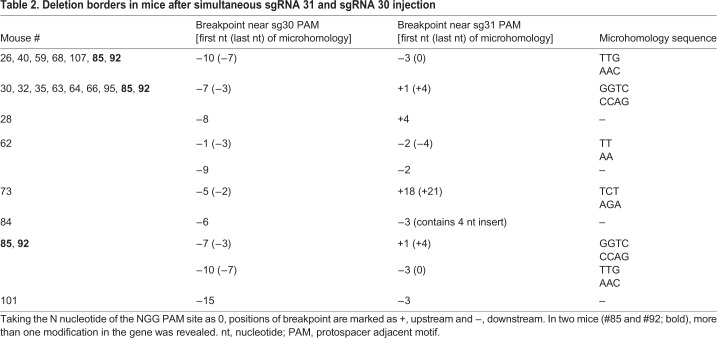
**Deletion borders in mice after simultaneous sgRNA 31 and sgRNA 30 injection**

For both sgRNA 30 and sgRNA 31, three probable off-target sites as predicted by CHOPCHOP online software (Table 3) were chosen for experimental assessment. In the genomes of all 19 newborn mice, these sites did not bear any mutations, as verified by the T7 endonuclease I (T7EI) test for all samples and direct Sanger sequencing of five random samples for each off-target site (data not shown).

**Table 3.**
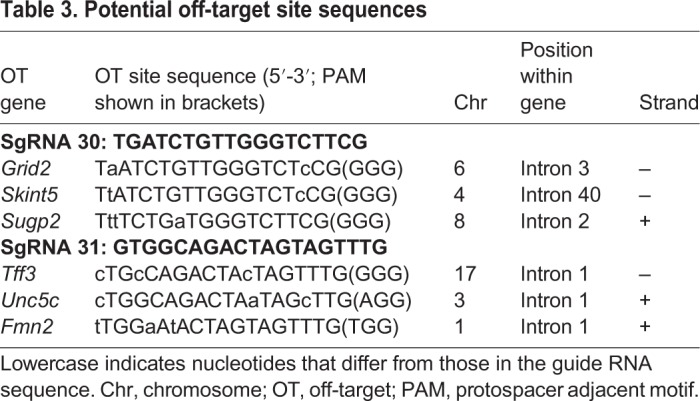
**Potential off-target site sequences**

### Analysis of the mouse phenotype

**Fig. 3.**
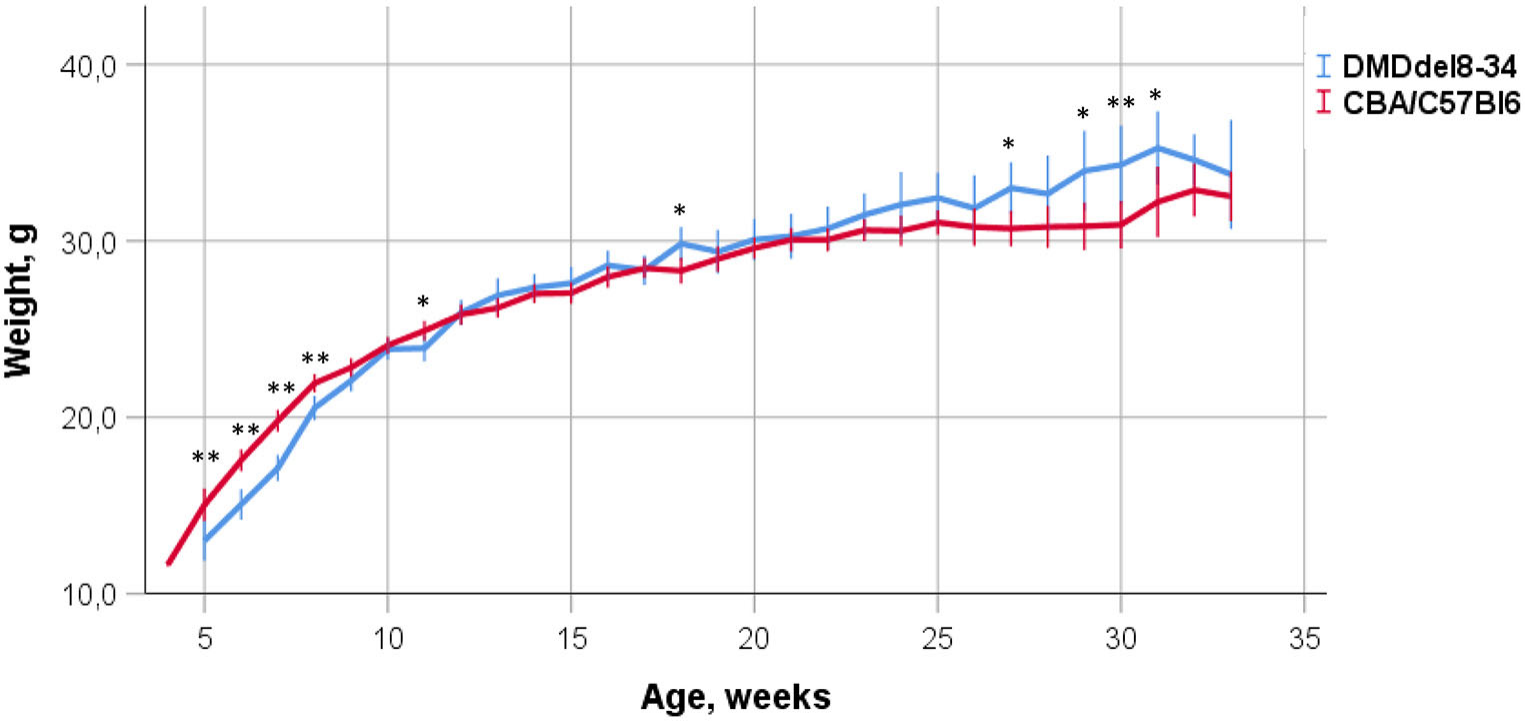
**Effect of the introduced mutation on body weight gain.** DMD^del8-34^ mice (blue) and their control CBA×C57BL/6 (red) male littermate mice were regularly weighed from the age of 5 weeks to 33 weeks of age. Each data point is the mean±2 s.e.m. (*n*=21-93; *P<0.05, **P<0.01, one-way ANOVA).

Four female mice carrying the deletion of exons 8-34 in the *Dmd* gene (#35, #40, #64, #101) were used for the production of experimental and control animal cohorts. The mice were first bred with CBA×C57BL/6 (B6CBAF1) hybrid males, and the resulting F1 females that were heterozygous for the introduced *Dmd* mutation were further bred with CBA×C57BL/6 males. The male progeny of this cross that inherited the *Dmd* mutation (DMD^del8-34^ mice) were used for the analysis of phenotypical changes caused by the mutation, whereas their male littermates not bearing the mutations were used as wild-type controls. The animal weight dynamics were assessed from weaning until week 34 in comparison with the wild-type control group. Through the first 12 weeks, DMD^del8-34^ mice lagged behind the control mice in weight gain. These mice then reached the same average mass level as the control group mice before surpassing them in weight (Fig. 3).

**Fig. 4.**
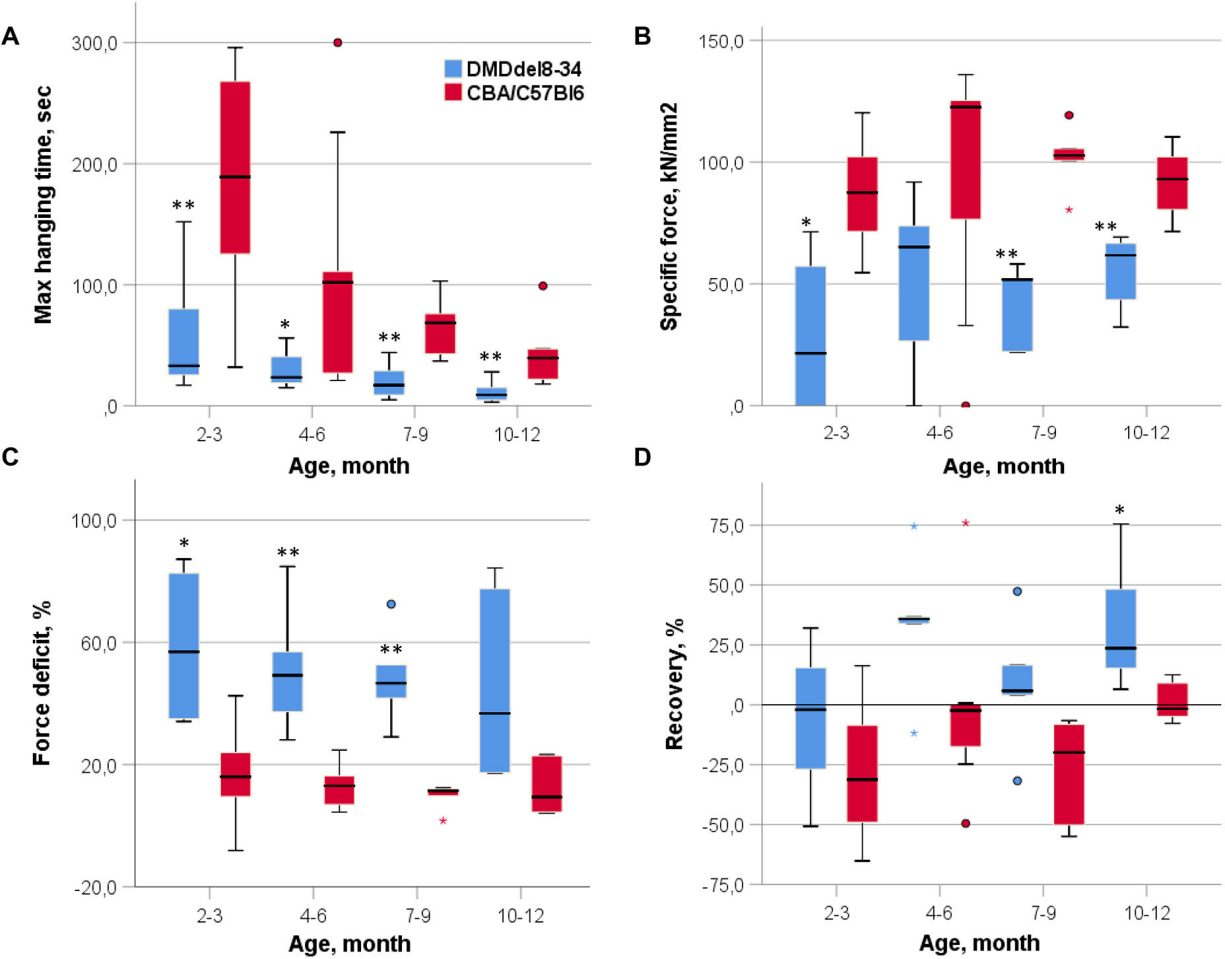
**Muscle force examinations.** (A) Performances of DMD^del8-34^ and wild-type control mice in the wire hanging test. Animals were tested at the indicated ages. Each session lasted 300 s, and mice were subjected to three trials. (B) Specific force comparison of DMD^del8-34^ and wild-type control mice. The TA contraction force was normalized to the muscle cross-sectional area. (C) Force deficit comparison of DMD^del8-34^ and wild-type control mice. The contraction force drop-off was assessed 1 min after the second muscle lengthening. (D) Performance of DMD^del8-34^ and wild-type control mice in the muscle recovery test. The recovery rate was assessed 15 min after the second muscle lengthening. The heavy black line inside each box marks the median of that distribution. The lower and upper box boundaries mark the 25th and 75th percentiles of each distribution, respectively. Whiskers represent 95% confidence intervals. Outliers are identified with a filled circle. Age groups were analyzed in pairs using the Mann–Whitney *U*-test for nonparametric data, *P<0.05, **P<0.01, *n*=6.

The wire hanging test was employed to assess animal muscle strength. The tibialis anterior (TA) muscle-specific force, force deficit after two lengthenings and muscle recovery were also calculated (Fig. 4). DMD^del8-34^ mice and their age-matched wild-type littermates differed significantly in both maximal hanging time (*P*-value=0.002, 0.021, 1.52E−4 and 0.009 for corresponding age groups in the hanging test, Fig. 4A) and muscle force deficit after two lengthenings in all age groups except those at 10-12 months (*P*-value=0.016, 0.001, 0.008 and 0.052, respectively, Fig. 4C). Specific force differed significantly between groups at 2-3 months, 7-9 months and 10-12 months (*P*-value=0.016, 0.08, 0.004, respectively, Fig. 4B) and did not reach significance at 4-6 months (*P*-value=0.097, Fig. 4B). The muscle recovery rate was significantly different for 10- to 12-month-old DMD^del8-34^ mice in comparison with those of wild-type mice (*P*-value=0.017, Fig. 4D).

**Fig. 5.**
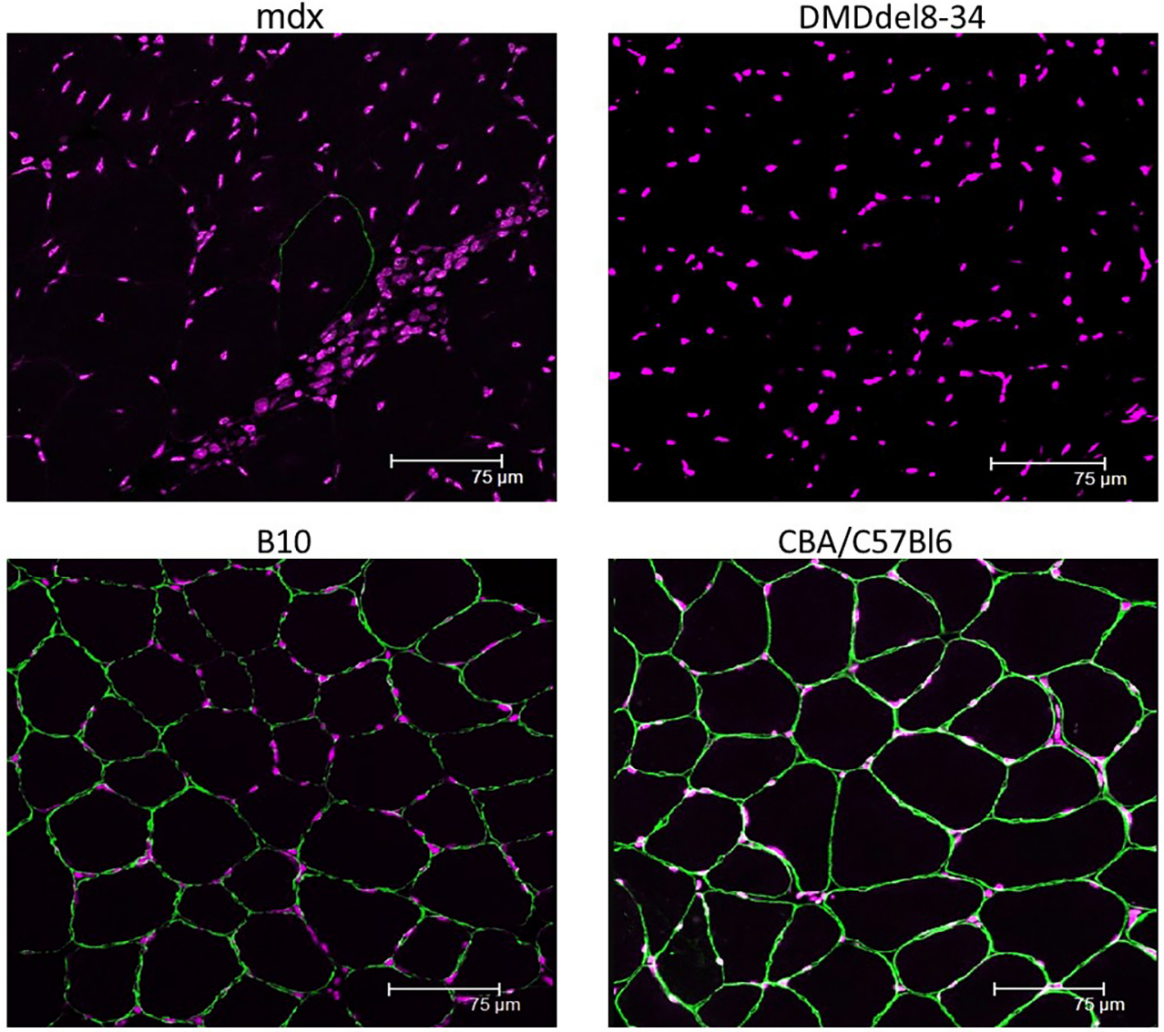
**Deletion of exons 8-34 in the dystrophin gene results in the absence of dystrophin protein in the sarcolemma of muscle cells.** Cross-sections through the TA muscle of *mdx* and DMD^del8-34^ mutant male mice, as well as those of their respective wild-type control C57BL/10ScSnJ (B10) and CBA×C57BL/6 mice, were immunostained with an antibody against the C-terminal amino acids of dystrophin (ab15227) and an Alexa Fluor 488 secondary antibody (green channel). The absence of dystrophin is evident in both DMD^del8-34^ and *mdx* mutant mice. Scale bars: 75 µm.

**Fig. 6.**
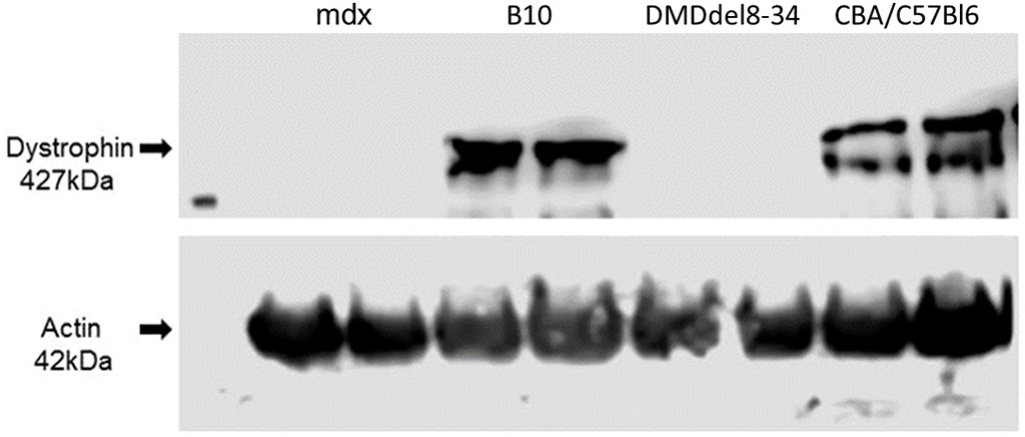
**Deletion of exons 8-34 of the *Dmd* gene results in the absence of the full-length dystrophin protein from the muscles of male mutant mice.** Western blot analysis of protein lysates from muscles of DMD^del8-34^ and *mdx* mutant male mice, as well as those of their respective wild-type control C57BL/10ScSnJ (B10) and CBA×C57BL/6 mice, using an antibody against the dystrophin protein (ab154168). Equal loading of protein lysates was confirmed by reprobing the membrane with an antibody against alpha-actin. The absence of full-length dystrophin is evident in muscle lysates of both DMD^del8-34^ and *mdx* mutant mice.

*Mdx* mice and their genetic background strain C57BL/10ScSnJ mice (B10 in figures) were also subjected to experiments to compare the effects of two different mutations of the *Dmd* gene. Similar to *mdx* mice, full-length dystrophin was not detected in the muscles of DMD^del8-34^ mutant male mice using an antibody that recognizes the C-terminal epitope of the protein by immunohistochemistry (Fig. 5) or western blotting (Fig. 6).

**Fig. 7.**
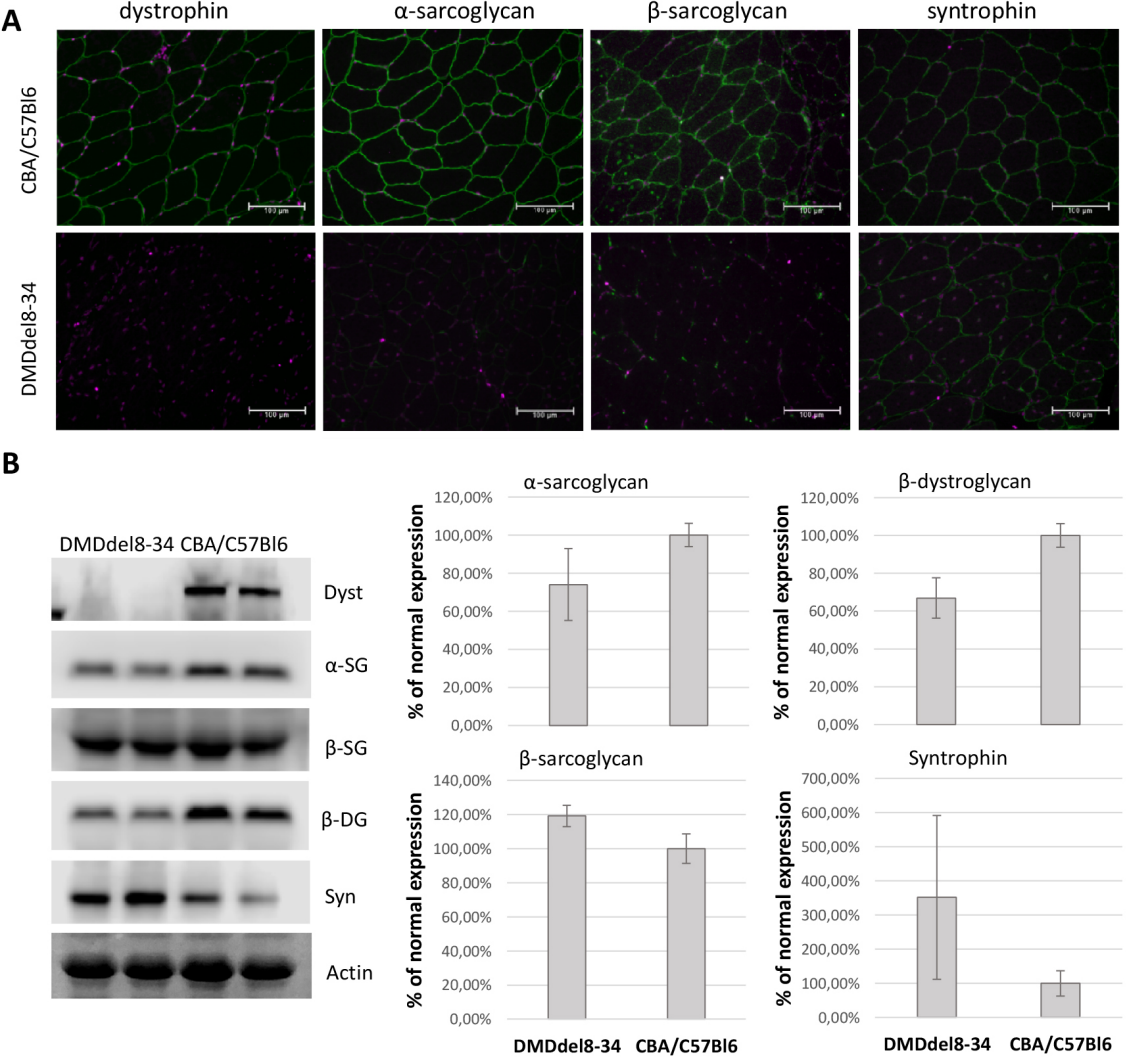
**Analysis of DAGC component expression levels in the muscles of DMD^del8-34^ mice in comparison with that in wild-type controls.** (A) Cross-sections through the TA muscle of DMD^del8-34^ mutant male mice, as well as those of their wild-type control CBA×C57BL/6 mice, were immunostained with an antibody against DAGC proteins (green). Nuclei were counterstained with To-Pro-3 Iodide (pseudocoloured magenta). Scale bars: 100 µm. (B) Total muscle lysates of TA muscles from DMD^del8-34^ and CBA×C57BL/6 mice were analyzed by western blot using an antibody against DAGC proteins. Alpha-actin was used as a loading control. Representative images of three independent experiments are shown. Graphical representation of DAGC component expression in TA muscle of DMD^del8-34^ in comparison with that in wild-type control mice from blots is shown to the right. Downregulation of alpha-sarcoglycan and beta-dystroglycan, but upregulation of beta-sarcoglycan and syntrophin total protein level are shown. Bar charts show the mean±2 s.e.m.

Immunostaining and western blot analyses were performed to evaluate the DAGC component expression levels in sarcolemma and whole-muscle lysates. As shown in Fig. 7A, significantly reduced levels of alpha- and beta-sarcoglycans and lower levels of syntrophin were detected in the sarcolemma of TA cross-sections harvested from mutant mice compared with those from the wild-type control. The total protein levels of alpha-sarcoglycan and beta-dystroglycan were reduced, whereas those of beta-sarcoglycan and syntrophin were elevated (Fig. 7B).

**Fig. 8.**
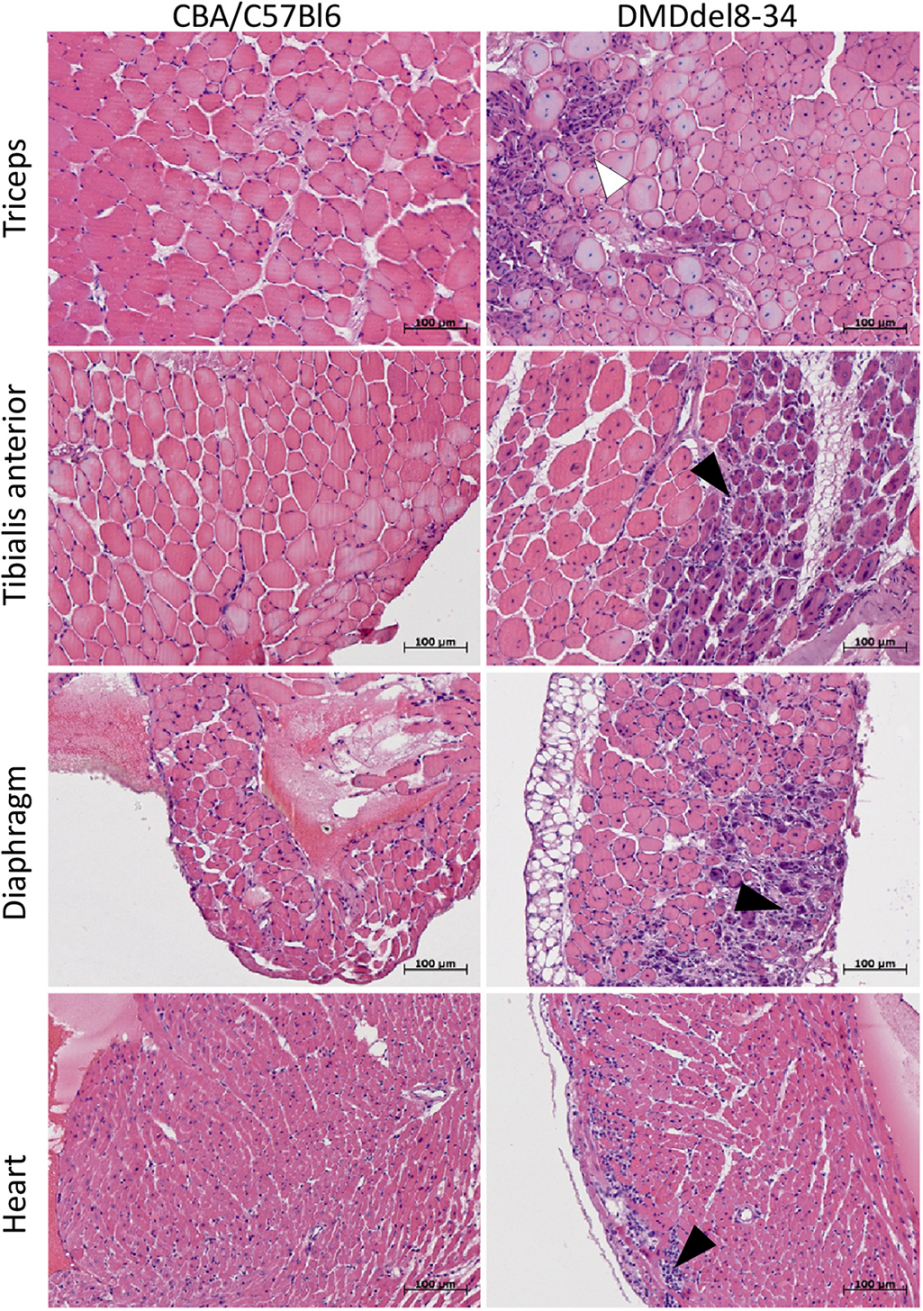
**Representative images of H&E-stained muscles.** Muscle fibres of control CBA×C57BL/6 mice had equal diameter; the nuclei are located on the periphery of muscle fibres. DMD^del8-34^ mice had variation in fibre size; over half of the myofibres had centrally located nuclei. H&E staining also revealed signs of inflammation, necrosis and regeneration in the skeletal muscles and diaphragm of DMD^del8-34^ mice. The white arrowhead indicates the regeneration zone (small myofibres with a more basophilic cytoplasm); the black arrowheads indicate infiltrating inflammatory cells. Scale bars: 100 µm.

Haematoxylin and eosin (H&E) staining was used to observe the overall histopathology of muscles. Representative images from 12-week-old mice are shown in Fig. 8. Histological analysis showed myofibre damage and many fibres with centrally located nuclei, which indicated degeneration and subsequent regeneration. The identified morphological features of DMD^del8-34^ muscles included prominent myofibre necrosis, the presence of infiltrating inflammatory cells and fibrotic scars replacing damaged myofibres. The number of centronucleated fibres in skeletal muscles was twofold higher than that in diaphragms (70-80% and 30-40%, respectively). Examination of sections showed that skeletal muscles (TA and triceps) and the diaphragm had more prominent dystrophic pathomorphology than the heart.

**Fig. 9.**
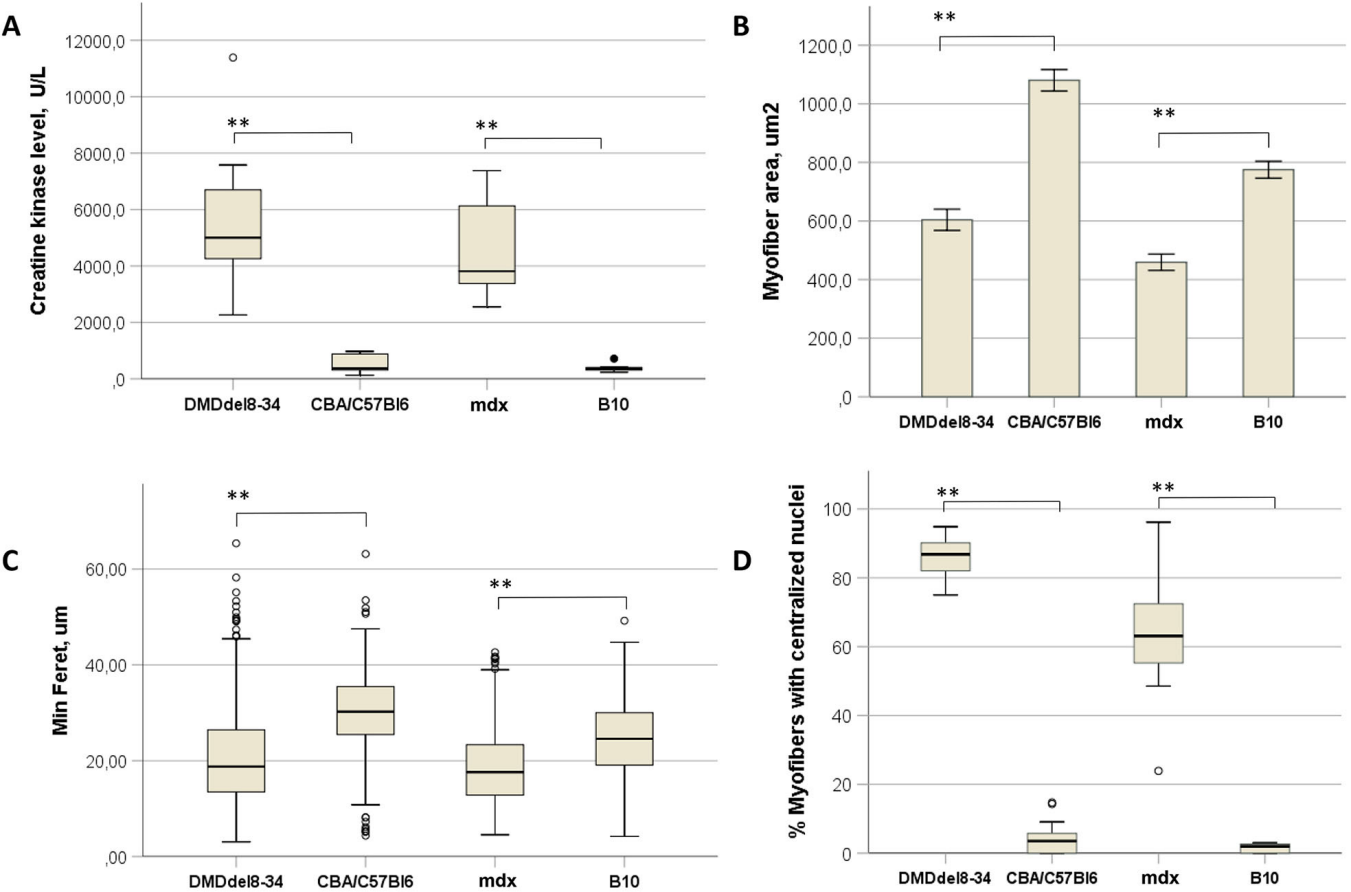
**Histological and biochemical changes in the muscles of DMD^del8-34^ mutant mice.** (A) CK was measured in the blood sera of 12-week-old DMD^del8-34^ and *mdx* mutant male mice, as well as in that of their respective wild-type control C57BL/10ScSnJ (B10) and CBA×C57BL/6 male mice. (B-D) The myofibre cross-sectional area (B), minimal Feret diameter (C) and percent of myofibres with centralized nuclei (D) were analyzed in the cross-sections through the TA muscles of 12-week-old DMD^del8-34^ and *mdx* mutant male mice, as well as in those of their respective wild-type control C57BL/10ScSnJ (B10) and CBA×C57BL/6 mice male mice. The heavy black line inside each box (A,C,D) marks the median of that distribution. The lower and upper box boundaries mark the 25th and 75th percentiles of each distribution, respectively. Whiskers represent 95% confidence intervals. Outliers are identified with a filled circle. Groups were compared in pairs using the Mann–Whitney *U*-test for nonparametric data. **P<0.01, *n*=6 (A); *n*=800 per group (C,D). Bar chart (B) shows the mean±2 s.e.m. of measured parameters. **P<0.01, using one-way ANOVA with Fisher's LSD post hoc test. *n*=800 per group.

Analysis of muscle morphology on histological sections of the TA revealed that the median muscle fibre cross-sectional area (CSA; 604 µm^2^) and median minimal Feret diameter (20.7 µm) were significantly smaller (*P*-value=1.2E–64 and 1.42E–85, respectively; Fig. 9B and C) in DMD^del8-34^ mice than in wild-type mice. Central nucleation was detected in 86% of myofibres from DMD^del8-34^ mice in comparison with 4% in those of control mice (Fig. 8, Fig. 9D).

The CK content in the blood serum of DMD^del8-34^ mice was ∼10× higher than that in wild-type mice (*P*-value=0.001), and this level was not significantly different from the CK in the blood serum of *mdx* mice (*P*-value=0.445, Fig. 9A).

## DISCUSSION

A novel, *de novo* mutation in the *DMD* gene leading to a frameshift deletion of exons 8-34 was found in a Russian patient with muscular dystrophy. According to the theoretical translation of the corresponding shortened mRNA, the presence of only N-terminal ABD is possible in the muscle cells of the patient (Fig. 2C). We produced a new mouse strain replicating this *Dmd* gene mutation and demonstrated that male mice carrying this mutation recapitulated several key features of DMD pathology. Thus, this mouse strain represents a good model of this type of DMD and can be beneficial for designing a patient-specific therapy based on exon skipping. The frequency of mutations that can be treated by simultaneously skipping exons 6 and 7 was estimated as 3% (Aartsma-Rus et al., 2009). Our data correlate well with published statistics, as we identified 18 patients with such mutations in the Russian DMD registry containing ∼600 patients (Table 4).

**Table 4.**
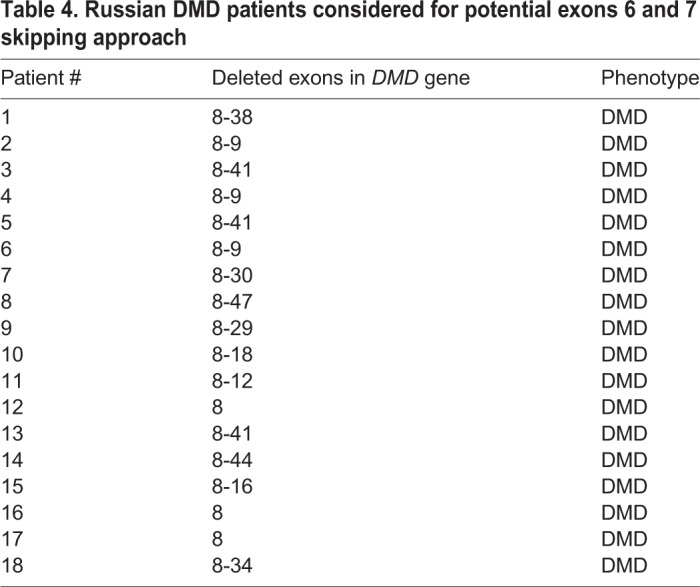
**Russian DMD patients considered for potential exons 6 and 7 skipping approach**

Contrary to the viral delivery of shortened forms of dystrophin or cell therapy, the exon skipping approach requires test models for each mutation type. Today, only a few mouse models are available for such tests, and none is suitable for modelling exon skipping therapy affecting ABD domains and flanking regions, i.e. exons 1-7 of the *Dmd* gene. Thus far, only one murine model involving mutations in ABD has been generated, and this model represents a *Dmd* first exon duplication. In this case, successful exon skipping can completely restore the reading frame (Vulin et al., 2015). This model cannot be used to test for the skipping of ABD-coding exons.

Two clinical cases of deletions in ABD listed in the Leiden database are completely different, as one patient had severe symptoms and was described as a DMD patient, whereas the second patient had BMD (Yang et al., 1994; Basak et al., 2006; White and den Dunnen, 2006). Moreover, deletion of exon 5 results in severe DMD symptoms, although without a frameshift (Toh et al., 2016). Deletion of exons 3-9 was shown to be asymptomatic or lead to the BMD phenotype in 11 of 15 patients (Nakamura et al., 2016). Four cases of in-frame deletions containing exons 6 and 7 listed in the Universal Mutation Database (UMD)-DMD database (6-10, 6-12 and 6-13 exons) are characterized as DMD or intermediate muscular dystrophy (Béroud et al., 2000). Based on these conflicting data, we cannot conclude that the skipping of exons 6 and 7 is a suitable treatment strategy for patients with mutations similar to that described in our manuscript.

In the case of successful skipping of exons 6 and 7, mRNA translation will result in a short dystrophin protein that lacks part of the ABD (CH2) and part of the rod domain, including hinges 1-2 and spectrin repeats 1-12, but will contain repeats 16 and 17, which represent the nNOS-binding site, cysteine-rich domain and C-terminal domain (Allen et al., 2016); a schematic of the resulting protein is shown in Fig. 2D. Thus, further experiments should explain the roles of absent domains in dystrophin function and possible enhancements of DMD treatment strategies.

Our new mouse model of DMD shows the main biochemical and histological symptoms of dystrophy, such as increased CK content and number of centralized nuclei, and decreased average minimal Feret diameter and myofibre CSA. In addition, extensive calcification, fibrosis, immune cell infiltration and reduced levels of DAGC components on the sarcolemma were detected. All of the symptoms were similar to those of *mdx* mice of the same age. Dystrophin deficiency and its consequences influence the locomotor activity of model mice. Lowered muscle strength and endurance, force deficit and longer recovery time were shown for different age groups of model animals in comparison with those of their age-matched wild-type littermates. The weight dynamics of mutant mice differed from those of control mice and were similar to those of *mdx* mice. The DMD^del8-34^ mouse body weight was lower in postnatal development but reached that of the wild-type level at 9-10 weeks of age, and the mutant mice then outgrew their counterparts. In addition, a higher diversity in body weight was characteristic of the mutant mice. Similar dynamics have been shown for *mdx* mice, as they were shown to gain body weight comparably to wild-type mice by 11 weeks of age (Durrant et al., 2012).

The mouse model described herein represents the largest deletion of the *Dmd* gene created in animals using the CRISPR/Cas9 tool (Wells, 2018). The CRISPR/Cas9 genome editing strategy is of major interest as a potent tool for the creation of new models and gene recovery to cure various pathogenic conditions. Two mechanisms of double-strand break repair exists in cells, homology-directed repair (HDR) and non-homologous end-joining (NHEJ). Deletion borders in the analyzed mutant mice indicate NHEJ participation in double-strand break reparation after *Streptococcus* *pyogenes* *Cas9* mRNA/gRNA injection into zygotes. Moreover, two types of microhomology sequences were identified in a majority of samples (14 of 19, Table 2). These data correlate with data published for *Arabidopsis thaliana* (Shen et al., 2017) and tracheal epithelial (CFTE) mammalian cell line experiments (Hollywood et al., 2016). For different applications, predicting the exact deletion borders after the simultaneous injection of two guide RNAs in complex with Cas9 can be important. The results reported herein suggest that assuming all known data from *in vivo* Cas9 experiments can help to make such a prediction.

**Fig. 10.**
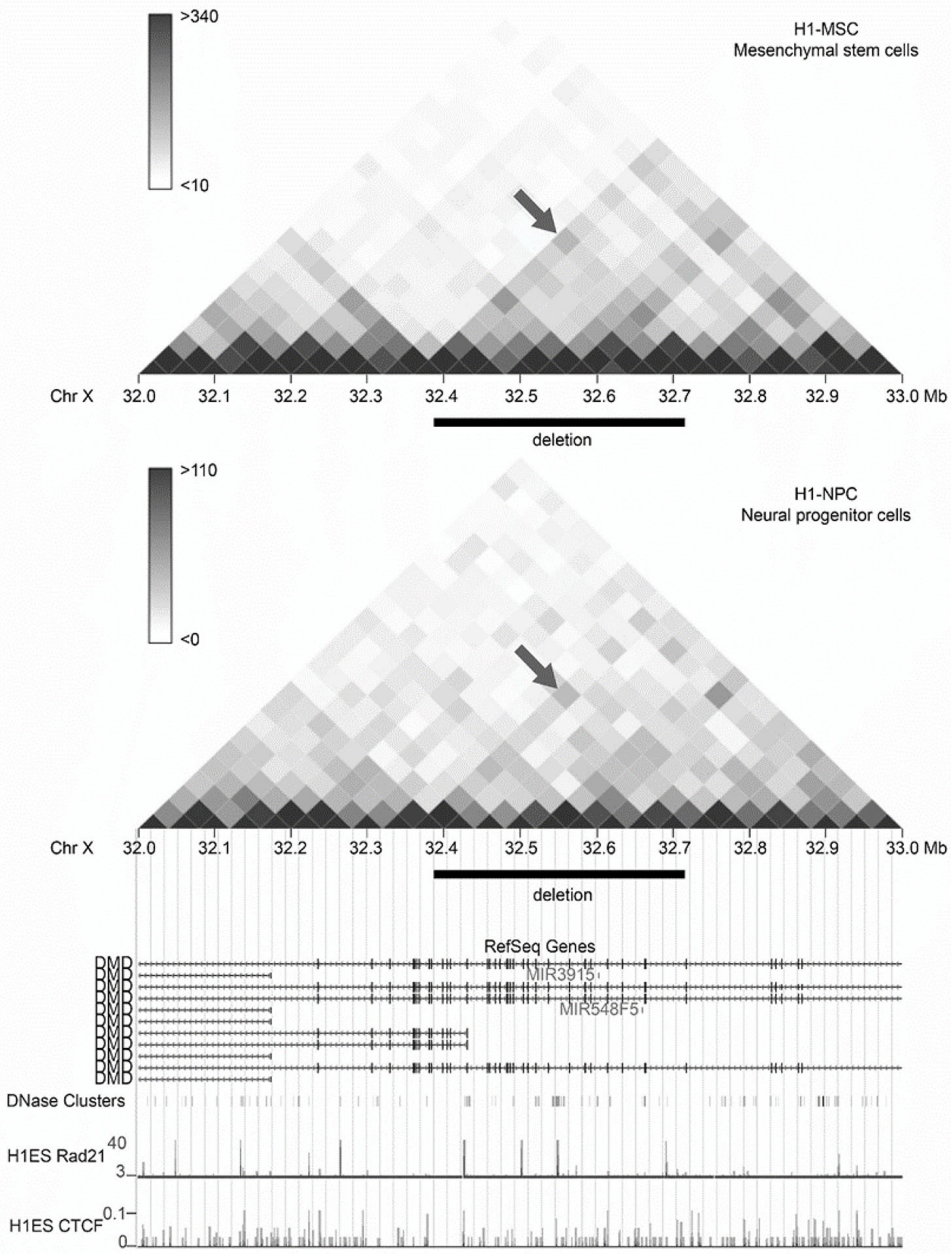
**Chromatin interactions** **in the human *DMD* gene.** Heat map visualization of Hi-C interactions in the 1 Mb region of the *DMD* gene with 40 kb resolution (data from YUE Lab interactive Hi-C browser; promoter.bx.psu.edu/hi-c/view.php/). The black rectangle shows the location of the deletion. The arrows point to a spot that indicates an increased number of contacts between loci homologous to those flanking the deletion. RefSeq, DNase, CTCF and Rad21 data are also shown.

The spatial organization of the genome has recently gained substantial attention as a factor that influences the patterns of chromosomal rearrangements and translocations (Fudenberg et al., 2011; Zhang et al., 2012). By exploring publicly available long-range chromatin interaction (Hi-C) data [3D Genome browser: visualize Hi-C, ChIA-PET, Capture Hi-C data; http://promoter.bx.psu.edu/hi-c/ (accessed: 21 November 2017)] for the 1 Mb region of ChrX encompassing the deletion, we found that in several human cell lines, including mesenchymal and neural stem cells, chromosomal sites flanking the deletion preferred to interact with each other (Fig. 10). It thus may be hypothesized that the location of the two sites in spatial proximity may predispose them to deletion. Future research may help to elucidate the molecular mechanisms that underlie the formation of the deletion.

## MATERIALS AND METHODS

### Ethics statement

All animal studies were conducted according to experimental practices and standards approved by the Ethics Committee of the Institute of Gene Biology, Russian Academy of Sciences. All mice were maintained under a controlled photoperiod (lights on 07:00-19:00). Food and water were provided *ad libitum*.

### Patient

The patient was identified and enrolled at the Russian Children’s Neuromuscular Center in Moscow, Russia. Two family members, the 8-year-old boy who presented with the symptoms of DMD and his mother, took part in the study. Informed consent was obtained from the participants. The diagnosis of DMD was made on the basis of symptoms, EMG data and blood CK levels and was confirmed by the MLPA genetic assay.

### Cell lines

Cell lines used in this work were recently authenticated using short tandem repeat DNA profiling (Gordiz) and tested for contamination using PCR.

### Fibroblast transdifferentiation into myoblast

Lentivirus production and transdifferentiation experiments were performed based on protocols described by Kabadi and colleagues (Kabadi et al., 2015). Briefly, 0.5 million HEK293T cells were plated per six-well plate. The following day, cells were transfected with 1 μg of transfer vector (LV-TRE-VP64 human MyoD-T2A-dsRedExpress2, a gift from Charles Gersbach, Addgene plasmid #60629), 0.3 μg of pMD2G (Addgene plasmid #12259) and 0.5 μg of psPAX2 (Addgene plasmid #12260) using linear polyethylenimine transfection reagent; the media was changed 12-14 h post-transfection. At 48 h after the media change, the viral supernatant was pooled and passed through a 0.45 μm filter. Primary fibroblasts from the patient and healthy male control were transduced with a MyoD-encoding lentivirus in the presence of 4 μg/ml polybrene. Cells were selected in 2 μg/ml puromycin to obtain a pure population of transduced cells. Cells were expanded in standard growth medium supplemented with puromycin. Selected cells were grown to confluence, and MyoD transgene expression was induced by supplementing the medium with 3 μg/ml doxycycline. Cells were given fresh media supplemented with doxycycline every 2 days. Samples were collected for analysis after 14 days of doxycycline induction.

### Mutation analysis

Total genomic DNA was extracted from the patient's venous blood using the QIAamp DNA mini kit (Qiagen). The MLPA analysis was performed using the SALSA MLPA probe mixes P034 DMD-1 and P035 DMD-2 (MRC-Holland). For library preparation, 1 µg of DNA was fragmented using a Covaris S220 instrument. End repair, adenylation, adapter ligation and PCR amplification were performed using reagents from the TruSeq DNA sample preparation kit (Illumina) according to the manufacturer's instructions. The library was quantified using qPCR primers I-qPCR-1.1 (5′-AATGATACGGCGACCACCGAGAT-3′) and I-qPCR-2.1 (5′-CAAGCAGAAGACGGCATACGA-3′) diluted to 10 pM and sequenced on a HiSeq2000 instrument (three lanes of v.3 flowcell; Illumina) with a read length equal to 101 from each end of the fragment. FASTQ files were generated using CASAVA-1.8.2 (Illumina), and 52.5 Gb of total raw data were obtained. Genomic analysis was performed using the Science Advice-pipeline (Research Center for Genetic Medicine, Science-Advice.com).

To study whether the patient's mutation was inherited from his mother, a set of four PCR primers were designed. The first pair of primers, LbF and LbR, were designed to amplify an 857 bp fragment overlapping the left border of the deletion in the patient's genome, and the second pair, RbF and RbR, were designed to amplify an 823 bp fragment overlapping its right border. For these two primer pairs, amplification is observable only in the absence of the deletion. The combination of the LbF and RbR primers yielded a readthrough product of 815 bp in the case of deletion, and no amplification product could be generated from the normal allele because of the distance between these primers (∼0.3 Mb). The PCR amplification programme was as follows: an initial denaturation step for 5 min at 94°C; 30 cycles of 30 s at 94°C, 30 s at 58°C and 1 min at 72°C; and a final step of 7 min at 72°C. PCR was performed using the GenePak PCR core kit (Izogen Lab Ltd, Russia). The electrophoretic detection of PCR products was performed using a 2% agarose gel with an intercalating dye. The direct sequencing of PCR-amplified products was performed at Evrogen.

### *Cas9* mRNA *in vitro* transcription

The Cas9 expression construct pET28a/Cas9-Cys, a gift from Hyongbum Kim (Addgene plasmid #53261), was linearized and transcribed *in vitro* using the mMessage mMachine T7 kit (Ambion) followed by phenol-chloroform extraction.

### sgRNA design and synthesis

sgRNAs were selected using CHOPCHOP online software (Montague et al., 2014; Labun et al., 2016) in the 7th and 34th introns of the mouse *Dmd* gene (*Dmd* GenBank ID: NC_000086.7). For sgRNA synthesis, two partially complementary oligonucleotides (oligos) were used: sgR, encoding the T7 promoter, and sgF, encoding the guide RNA sequence. Both oligos were synthesized by Evrogen. For template amplification, oligos were combined in an equimolar ratio and polymerized in a T100 thermocycler (Bio-Rad) using the following programme: 95°C for 1 min; 30 cycles of 95°C for 30 s, 65°C for 30 s and 72°C for 30 s; and 72°C for 5 min. The GenePak PCR core kit was used (Izogen Lab). To purify the template from the reaction mix, the Clean Up kit (Evrogen) was used, and the sgRNA was then transcribed from the T7 promoter *in vitro* using the RiboMAX Express kit (Promega). Reaction components were removed by phenol-chloroform extraction followed by isopropanol precipitation. Synthesized sgRNAs were dissolved in nuclease-free water. The concentration was determined using the Qubit RNA HS kit (Thermo Fisher Scientific), and the sgRNAs were stored at −70°C before use.

We also used the SureGuide Cas9 programmable nuclease kit (Agilent) to test freshly synthesized sgRNA activity *in vitro*. As a template for the new guide, a target region containing plasmid DNA was used. The target region for each guide was amplified and ligated into vector pTZ57R/T using an InstaClone PCR cloning kit (Thermo Fisher Scientific).

### Microinjections into the pronucleus of zygotes, embryo cultivation and transfer

In immature female mice (C57BL/6×CBA) weighing 12-13 g, superovulation was induced by administrating 5 units of pregnant mare serum gonadotropin (Folligon, Intervet International) and 5 units of human chorionic gonadotropin (hCG, Pregnil, N.V. Organon) 46-48 h later. After injection, the female mouse was immediately placed in a cage with a male mouse for mating. Ovulation occurred within 11-14 h after hCG injection. Fertilized eggs were surgically washed out within 12-13 h after copulation, i.e. 25-27 h after hCG injection. The two pronuclear-stage zygotes were placed in a chamber consisting of two coverslips with one fixed above the other such that the upper and lower droplet edges of the M2 medium (MTI-GlobalStem) were flat and parallel. Pronuclei were visualized using a differential interference contrast microscope (Axiovert 200, Carl Zeiss). The microinjection needles were made from G100 glass capillaries (Narishige) on a P97 puller (Shutter Instruments) and holder capillaries were made from GD1 capillaries (Narishige) on a PC-10 puller (Narishige) and MF-900 Microforge (Narishige). For microinjection, guide RNA (20 ng/µl) was mixed with *Cas9* mRNA (50 ng/µl) or Cas9 protein (0.1 pM; New England Biolabs) in TE buffer [10 mM Tris-HCl (pH 7.4); 0.1 mM EDTA]. The components were mixed immediately before microinjection and incubated for 5 min at 37°C. The resulting genome-editing complex was microinjected into the male pronucleus of mouse zygotes. After microinjection, the zygotes were cultured for 2-3 h in a CO_2_ incubator (150 IGO, Thermo Electron Corporation) at 5% CO_2_ and 100% humidity and then assessed visually; those that were in satisfactory condition were transferred to recipient mice or left for 3 days in KSOM buffer (MTI-GlobalStem) to form a blastocyst. Cultivation took place in Petri dishes (35 mm in diameter) in 50-60 μl droplets; the top of the droplets was covered with embryo-tested light mineral oil (Merck KGaA). All the preparatory procedures were performed in a laminar flow hood.

### Total blastocyst DNA preparation and PCR amplification of the target fragment

Under a microscope, each embryo in 1 µl of buffer was sequentially transferred through three drops of PCR-quality water (Evrogen) using an automatic pipette and filtered tips, and placed in a 1 μl volume drop on the wall of a 0.2 ml tube; 1 μl of water from the last drop was used as the PCR negative control. When necessary, samples at this stage were frozen until further analysis and stored at −20°C. Blastocysts were lysed via proteinase K processing with detergent using the Sakurai et al. technique (Sakurai et al., 2008) with modifications. Briefly, 20 μl of buffer [proteinase K (125 μl/ml), Tris-HCl (100 mM, pH 8.3), KCl (100 mM), gelatine 0.02% and Tween-20 0.45%] was added to the test tubes for lysis. The tubes were incubated for 10 min at 56°C and then for 10 min at 95°C to inactivate proteinase. PCR analysis was performed immediately after sample preparation. The target region around the sgRNA recognition site was amplified using pairs of the forward and reverse primers sg30-sg35 (listed in Table S1) corresponding to the tested sgRNA. The GenePak PCR core kit (Izogen Lab) was used for PCR. The amplification programme was as follows: 95°C for 1 min; 40 cycles of 95°C for 30 s, 60°C for 30 s and 72°C for 30 s; 72°C for 5 min.

### Mutational analysis using bacteriophage T7 endonuclease I

PCR amplicons of target genomic regions were mixed with control non-mutated product, denatured and annealed at a temperature gradient from 95 to 25°C at a speed of 0.1°C/s. Hybridization products were digested using the T7EI enzyme (New England Biolabs) for 1 h at 37°C. Reaction products were separated by electrophoresis in an ethidium bromide-stained 2% agarose gel. The deletion boundaries and insertion sizes were determined by Sanger sequencing of the PCR products (Genome Center). The PCR primers used are listed in Table S1.

### Deletion detection on blastocysts

For deletion detection, a forward primer for the seventh intron (sg31F) and a reverse primer for the 34th intron (sg30R) were used, and a 700 bp product was amplified in only the case of a deletion. Control primers for the TET1 gene were used to verify the presence of DNA in the samples (primers are listed in Table S1). The GenePak PCR core kit (Izogen Lab) was used for PCR. The amplification programme was as follows: 95°C for 1 min; 40 cycles of 95°C for 30 s, 60°C for 30 s and 72°C for 30 s; 72°C for 5 min. Reaction products were separated by electrophoresis in an ethidium bromide-stained 2% agarose gel.

### Mouse genotyping

For mouse genotyping, tail biopsies were collected at the age of 2 weeks. A crude DNA sample was prepared using brief alkaline lysis; a small tail biopsy fragment was lysed in an alkaline lysis buffer [25 mM NaOH, 0.2 mM Na2-EDTA (pH 12)] for 1 h at 95°C, neutralized with 40 mM Tris-HCl (pH 5) and 2 μl of the resulting solution was used as a template for PCR. The primers, programme and detection methods were the same as those used for blastocysts.

### Off-target assay

Potential off-target sites of the sgRNAs were predicted using the E-CRISP online tool (Heigwer et al., 2014). The top three sites with off-target mutations were selected and used for PCR, T7EI and sequence analysis (Table 3). Geneious 8.1.3 (www.geneious.com) was used for sequence analysis. The primers used are shown in Table S1.

### Sample collection

Mice were euthanized by avertin overdose at the age of 12 weeks. Blood samples were collected from the heart with an insulin syringe and 30 G needle. Muscle samples were collected and either immediately frozen in liquid nitrogen for total protein extraction and western blot analysis or immobilized on Tissue-Tek O.C.T. Compound (Sakura Finetek) and frozen in liquid nitrogen-cooled isopentane for immunohistochemistry.

### Histological analysis of myofibres

Frozen 5 µm cross-cryosections of mouse TA muscles were fixed in 4% paraformaldehyde (PFA) for 20 min at room temperature and then incubated with FITC-conjugated wheat germ agglutinin (WGA; Sigma-Aldrich) and To-Pro nuclear dye (Invitrogen) for 30 min at room temperature. Three to five washes with phosphate-buffered saline (PBS) were performed after each step. All images were captured using a Leica SP2 confocal microscope. Ten photographs per muscle section were collected, and 800 myofibres were measured and analyzed per mouse using ImageJ software (Schneider et al., 2012) to determine the CSA, minimal Feret diameter and centralized nuclei count.

For H&E staining, dissected muscles were fixed in Bouin fluid and embedded in paraffin. Transverse sections (5-8 μm thick) were stained with H&E according to standard procedure (Slaoui et al., 2017). Images of stained sections were acquired with an Axiocam camera (Zeiss) at a magnification of 200×. The number of fibres was determined using ImageJ software and expressed as a percentage of the total fibre count.

### Immunohistochemistry

For immunohistochemistry, 5 μm cryosections of mouse TA muscles were fixed in 4% PFA in PBS and permeabilized with 0.1% Triton X-100 in PBS. Nonspecific binding was blocked with 5% bovine serum albumin and 0.1% Triton X-100 in PBS solution. Sections were stained overnight with rabbit polyclonal primary antibodies that recognized the C-terminal epitope of dystrophin (ab15277, 1:250, Abcam), other DAGC components (ab188873, ab189254, Abcam; sc-14176, Santa Cruz Biotechnology; all 1:500), and Alexa Fluor 488 goat anti-rabbit secondary antibody (ab150077, 1:1000, Abcam) for 1 h. Nuclei were counterstained with To-Pro dye. Antibodies and To-Pro dye were diluted in blocking buffer. Three 15 min washes with 0.1% Triton X-100 in PBS were included after each step. Section images were captured on the SP2 Leica microscope or DMI6000 Leica microscope.

### Western blot analysis

For western blot analysis, 100 mg of muscle was collected, physically disrupted and homogenized with a pestle in the presence of silicon dioxide powder and 1 ml of lysis buffer [75 mM Tris-HCl (pH 6.8), 10% SDS, 20% glycerol, 5% mercaptan, 0.001% bromophenol blue] and incubated at 95°C for 10 min to denature proteins. Each well on a precast mini-protean TGX 4-15% gel (Bio-Rad) was loaded with 5 μg of total protein. Proteins were transferred onto a nitrocellulose membrane using a standard wet transfer in Mini Trans-Blot Cell (Bio-Rad) using the manufacturer's protocol. Nonspecific binding was blocked by incubating the membranes in 5% dry milk in PBS for 3 h. Antibodies against target proteins (ab154168 1:1000, ab15277 1:400, ab188873 1:1000, ab189254 1:1000, ab15200 1:200, Abcam; NCL-b-DG 1:200, Novocastra; sc-14176 1:200, sc-304 1:200, Santa Cruz Biotechnology) or a rabbit monoclonal anti-actin antibody (A2103 1:10,000, Sigma-Aldrich) diluted in 1% dry milk in PBS were used for protein detection. Incubation with each antibody was carried out overnight at 4°C, followed by washes and incubation with corresponding horseradish peroxidase (HRP)-conjugated secondary antibodies (1/3000) for 1 h at room temperature and further washes in PBS. Enhanced chemiluminescence was used for the detection of protein bands, and images were obtained and analyzed on a C-DiGit Blot Scanner.

### Creatine phosphate kinase level measurement

Cardiac puncture under avertin anaesthesia was used to collect 250 μl of blood in an Eppendorf tube before sacrifice. Blood was left for 15 min at room temperature to promote clot formation before centrifugation (3500 ***g*** for 10 min at 4°C) and serum collection. Serum samples were stored at −80°C until analysis. CK activity was determined using the Creatine Kinase Activity Assay Kit (Sigma-Aldrich) and the Synergy 4 instrument (BioTek Instruments).

### Wire hanging test and muscle force examinations

The wire hanging test was performed according to the protocol described in Aartsma-Rus and van Putten (2014) on 2 mm wire installed 37 cm above a layer of bedding. We compared the 2-3 month, 4-6 month, 7-9 month and 10-12 month age groups of DMD^del8-34^ mice with their age-matched wild-type littermates. Each mouse was given three trials, and the maximum hanging time (the longest of the trials) was recorded and used for analysis.

The protocol for muscle preparation and lengthening was acquired from previous reports (Chan et al., 2007; Dellorusso et al., 2001) with little modification. Mice were anaesthetized with an initial intraperitoneal injection of 50 mg/kg Zoletil 100 (Virbac) and 5 mg/kg Rometar (Bioveta). The tendon of the TA was exposed by an incision at the ankle. The tendon was cut several millimetres distal to the end of the muscle and tied with a 4.0 nylon suture. The tendon and exposed muscle were kept moist by periodic applications of physiological saline. All experiments were conducted at room temperature (22-24°C). The foot and ankle of the mouse were fixed, and the tendon of the muscle was tied securely to the lever arm of a servomotor. All data were recorded using PowerGraph. The TA muscle was exposed to two stretches *in situ* at 100 Hz (frequency resulted in maximum tetanic force Po). The TA muscle was stimulated with 0.2 ms pulses via two electrodes that penetrated the skin on either side of the peroneal nerve near the knee. At the start of the experiment, the muscle was set to its optimum length (Lo) by finding the length that produced the maximum twitch force (Pt). Electrical stimulation started at time 0 ms. At 750 ms, the muscle was stretched at 1 mm/s until it was 15% longer than its optimum length, held at this length for 2 s, and then returned at the same rate to its original length. Stimulation was stopped at 5000 ms. A second lengthening contraction identical to the first was administered 10 s later, and the maximum isometric force was measured after 1 min, 5 min, 10 min and 15 min (P1, P5, P10 and P15, respectively). The maximum isometric force drop-off at 1 min corresponded to the muscle force deficit after the second lengthening [(Po-P1)/Po×100%]. The maximum isometric force exchange at 15 min corresponded to muscle recovery after the second lengthening [(P15-P1)/Po×100%]. After determination of the muscle's contractile properties, the muscle was excised, blotted on filter paper and weighed on an analytical balance. The total muscle CSA was approximated mathematically by dividing the muscle mass by the product of optimum fibre length (Lf) and 1.06 mg/mm^3^, the density of mammalian muscle. Lf was determined by multiplying Lo by the previously determined ratio of muscle length to fibre length (0.6 for TA muscle; Burkholder et al., 1994). Because absolute Po depends upon muscle size, Po values were normalized to muscle CSA (Po is divided by the calculated total muscle CSA) and expressed as the specific force (sPo; kN/mm^2^), where sPo=Po×(muscle mass/CSA).

### Statistical analysis

Data are mean±2 s.e.m. for normally distributed data. Differences identified between cohorts were determined using one-way ANOVA with Fisher's LSD post hoc test. For non-normally distributed data, the results are expressed as the median±95% confidence interval. Pairwise comparisons were performed using the Mann–Whitney *U*-test for nonparametric data. All data analyses were performed using SPSS statistics IBM software V.25.

## Supplementary Material

Supplementary information
