# Supplementary material for: CRISPR/Cas9-generated mouse model of Duchenne muscular dystrophy recapitulating a newly identified large 430 kb deletion in the human DMD gene
Source: Dis Model Mech. 2019 Apr 25;12(4):dmm037655. doi: 10.1242/dmm.037655 (PMC6505476; doi:10.1242/dmm.037655)
Supplement: Supplementary information [file dmm-12-037655-s1.pdf]

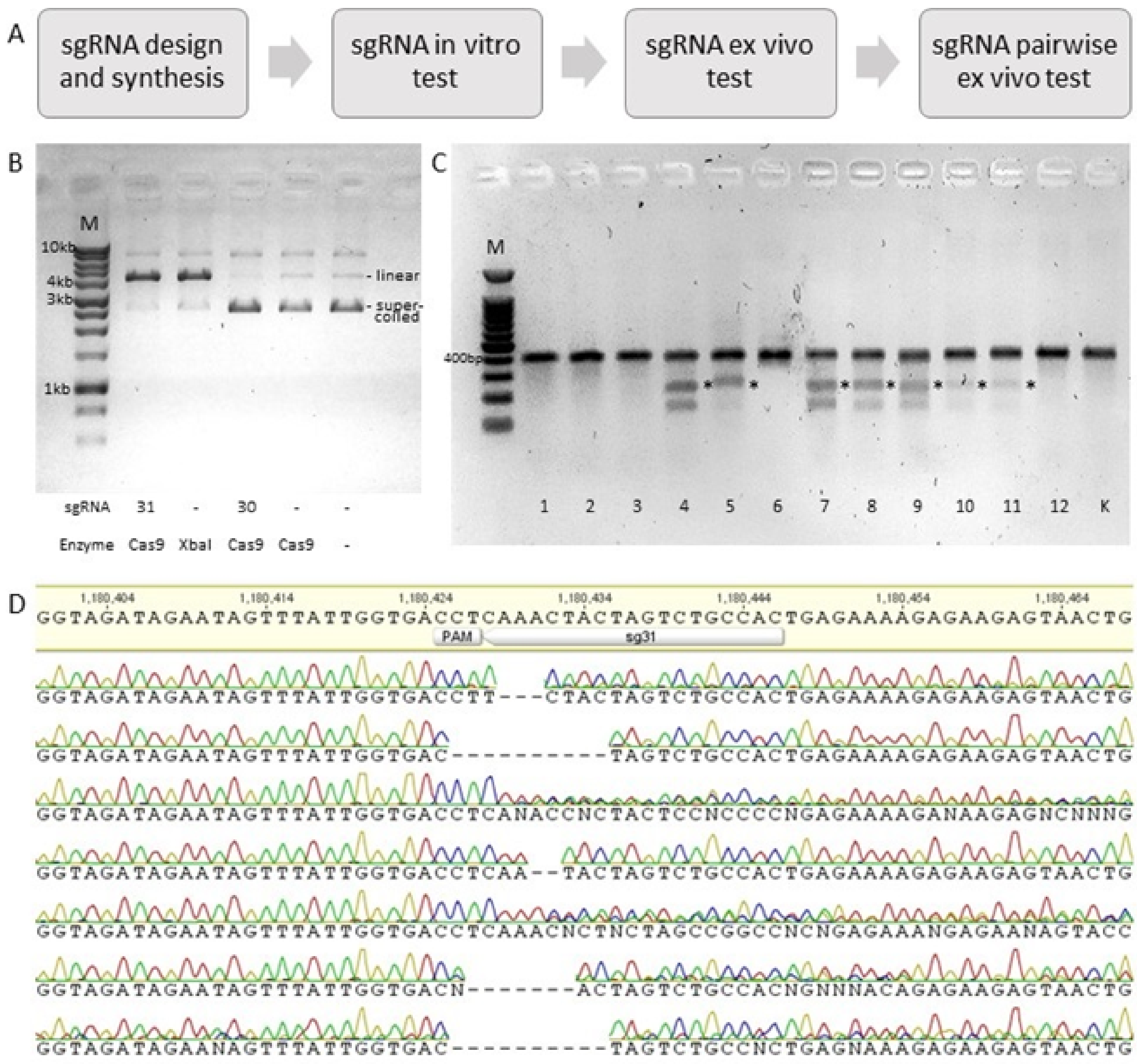

Figure S1. Guide RNA tests. A flowthrough of guide RNA testing is shown in panel A. The following test examples are for sg31. B. The cleavage capability of synthetic sgRNAs was assessed on a recombinant plasmid containing a PCR-amplified target fragment of the mouse *dmd* gene (plasmid length 4170 bp). This plasmid contained a single site for the XbaI restriction endonuclease, and digestion of the plasmid with this enzyme was used as a control for the efficiency of sgRNA-driven cleavage. At the same time, the selected plasmid could not be cleaved with the complex containing a nonspecific guide (sgRNA 30) or in the absence of sgRNA or both sgRNA and Cas9 protein. M-1 kb plus DNA ladder (NL001, Evrogen). C. The effectiveness of individual sgRNA was also tested on blastocysts; after direct injection of a

complex containing sgRNA and Cas9 mRNA, embryos were left in a CO_2 incubator before reaching the blastocyst stage. Then, the target region was amplified, and cleavage effectiveness was examined in the T7EI test. T7EI cleaves single-strand DNA, which is a result of partial complementarity of PCR products amplified from injected and noninjected embryos. Cleaved fragments are marked with asterisks and correspond to samples successfully modified by the Cas9-sg31 complex. M-100 bp plus DNA ladder (NL002, Evrogen). Direct Sanger sequencing of PCR products (D) confirmed the specificity of cleavage (products are mapped to the murine dystrophin gene).

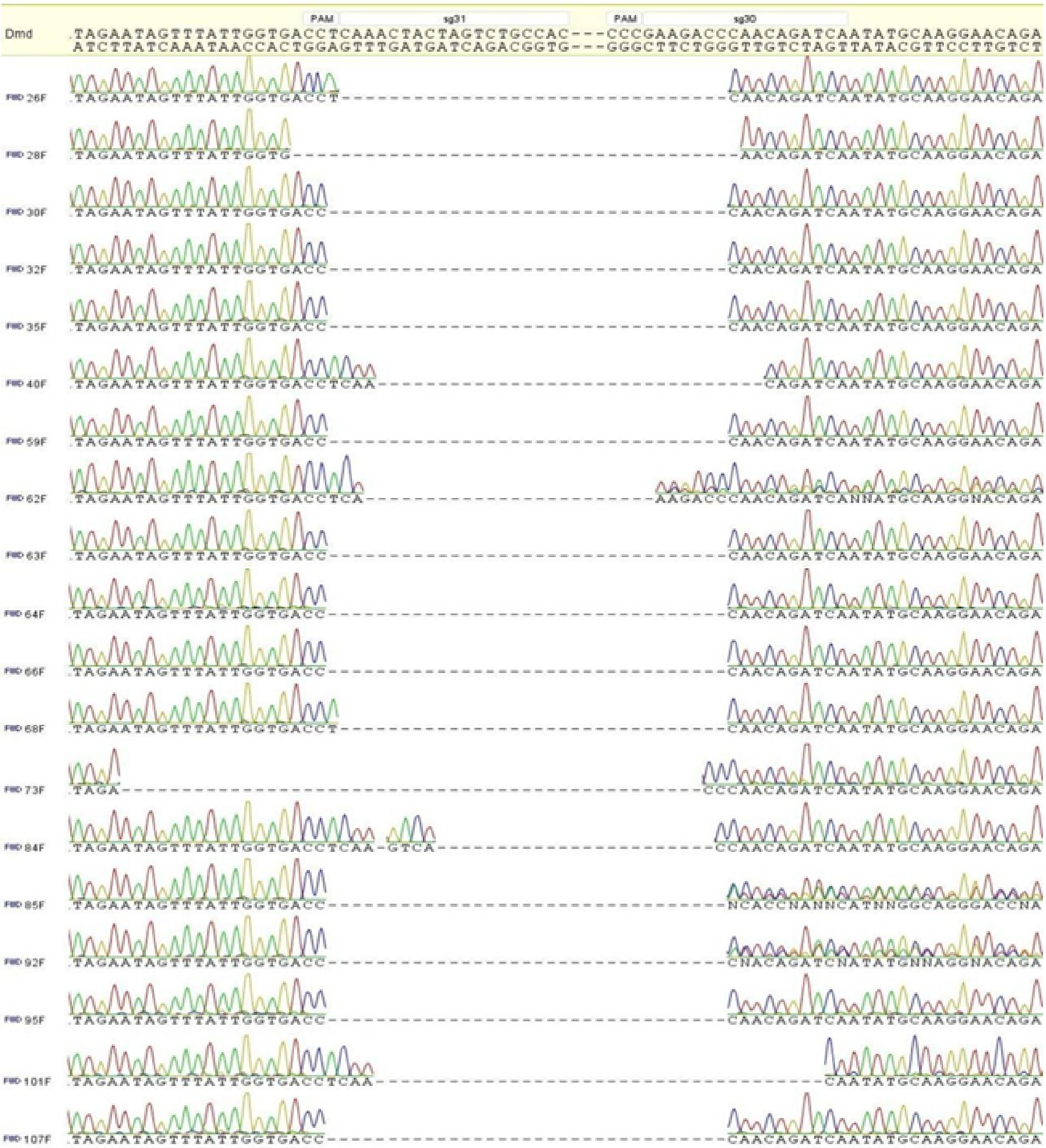

Figure S2. Sanger sequencing results of deletion borders in the *dmd* gene of mutant mice.

Disease Models & Mechanisms • Supplementary information

Table S1. Primers used for target site amplification for *in vitro* tests and deletion detection after blastocyst microinjections and mouse genotyping.

Target name	Forward primer, 5'-3'	Reverse primer, 5'-3'	Product size, bp
Lb	GCGTTTCATTCACTTTCTGGATGTG	AGTCACTTTAGGTGGCCTTGG	857
Rb	GCCAAGAGTTGCCTGAGAGGAAC	TGACAGTGAATAGTGACTCCAATGGC	813
sg30	ACTATTGTGGAACACAGCATACA	GAGAGAAAAGAGGCAGACTGTAG	607
sg31	TCAAACAAAAGGCAGAAGAGTAAG	GGTCCAAAGTAGGCCTCGTA	434
sg32	AGGCACATATAGTCAAGTTCAGTCA	TGCCATTCTGATCCTATTCATTTCC	730
sg33	GTTCTACTCTAAAACATCAGAGGCT	ACACATAGGACACATTCATGCAG	477
sg34	CAGTGCCCCACACACATACA	AGCAAAAGTTATTTTAGGGCATACT	505
sg35	CAGAGGTACTGGCATTTGGAAC	AAGTGGTATCCCCTTCTGCC	432
Tet	TTGTTCTCTCCTCTGACTGC	TGATTGATCAAATAGGCCTGC	460
Grid2	TCATATTATGGAGACCCCAACCA	GTGGCCAAGCAACTCCTTTT	360
Skint5	AAAGGGGACACCTGCTTCTG	GCCTACCTGGCTGTTTCAAGG	344
Sugp2	GAGCCATCTAAACGGCTGTC	GCTCCATGACAGGTAGGACT	573
Tff3	TGCAGAGGTTTGAAGCACCA	CCTGATGGCCAAGGGATGTT	406
Unc5c	GGGACTGGGTGTTTTTGCCT	TCTCTGCCTCACTGTCACCT	386
Fmn2	GGGCTCGTAGGGGTTCTTTAG	GGCTAATGGGTACATGGTCTC	265

Disease Models & Mechanisms • Supplementary information
